# Vaccination with a plasmid DNA encoding HER-2/neu together with low doses of GM-CSF and IL-2 in patients with metastatic breast carcinoma: a pilot clinical trial

**DOI:** 10.1186/1479-5876-8-53

**Published:** 2010-06-07

**Authors:** Håkan Norell, Isabel Poschke, Jehad Charo, Wei Z Wei, Courtney Erskine, Marie P Piechocki, Keith L Knutson, Jonas Bergh, Elisabet Lidbrink, Rolf Kiessling

**Affiliations:** 1 Department of Oncology and Pathology, Cancer Center Karolinska, Karolinska Institutet, Stockholm, Sweden; 2 Department of Surgery, Hollings Cancer Center, Medical University of South Carolina, Charleston, SC, USA; 3 Max-Delbrück Center for Molecular Medicine, Berlin, Germany; 4 Karmanos Cancer Institute, Wayne State University, Detroit, MI, USA; 5 Department of Immunology, College of Medicine, Mayo Clinic, Rochester, MN, USA

## Abstract

**Background:**

Adjuvant trastuzumab (Herceptin) treatment of breast cancer patients significantly improves their clinical outcome. Vaccination is an attractive alternative approach to provide HER-2/neu (Her2)-specific antibodies and may in addition concomitantly stimulate Her2-reactive T-cells. Here we report the first administration of a Her2-plasmid DNA (pDNA) vaccine in humans.

**Patients and Methods:**

The vaccine, encoding a full-length signaling-deficient version of the oncogene Her2, was administered together with low doses of GM-CSF and IL-2 to patients with metastatic Her2-expressing breast carcinoma who were also treated with trastuzumab. Six of eight enrolled patients completed all three vaccine cycles. In the remaining two patients treatment was discontinued after one vaccine cycle due to rapid tumor progression or disease-related complications. The primary objective was the evaluation of safety and tolerability of the vaccine regimen. As a secondary objective, treatment-induced Her2-specific immunity was monitored by measuring antibody production as well as T-cell proliferation and cytokine production in response to Her2-derived antigens.

**Results:**

No clinical manifestations of acute toxicity, autoimmunity or cardiotoxicity were observed after administration of Her2-pDNA in combination with GM-CSF, IL-2 and trastuzumab. No specific T-cell proliferation following *in vitro *stimulation of freshly isolated PBMC with recombinant human Her2 protein was induced by the vaccination. Immediately after all three cycles of vaccination no or even decreased CD4^+ ^T-cell responses towards Her2-derived peptide epitopes were observed, but a significant increase of MHC class II restricted T-cell responses to Her2 was detected at long term follow-up. Since concurrent trastuzumab therapy was permitted, λ-subclass specific ELISAs were performed to specifically measure endogenous antibody production without interference by trastuzumab. Her2-pDNA vaccination induced and boosted Her2-specific antibodies that could be detected for several years after the last vaccine administration in a subgroup of patients.

**Conclusion:**

This pilot clinical trial demonstrates that Her2-pDNA vaccination in conjunction with GM-CSF and IL-2 administration is safe, well tolerated and can induce long-lasting cellular and humoral immune responses against Her2 in patients with advanced breast cancer.

**Trial registration:**

The trial registration number at the Swedish Medical Products Agency for this trial is Dnr151:785/2001.

## Background

The proto-oncogene HER-2/neu (Her2) is overexpressed in a number of malignancies including breast, ovarian, cervical and renal carcinoma [1,2] and represents an attractive therapeutic target. Trastuzumab (Herceptin), a recombinant humanized monoclonal antibody binding Her2, induces durable objective clinical responses and/or improved time to relapse when administered in the adjuvant setting in women with Her2-expressing breast cancer as a single agent or in combination with chemotherapy [3-7]. However, trastuzumab was shown to be therapeutically ineffective in a proportion of patients and alternative strategies targeting their tumors are urgently needed [8,9].

Active specific immunotherapy, such as plasmid DNA (pDNA) vaccination, is an alternative approach to antibody therapy and several properties make Her2 a promising tumor vaccine candidate [10,11]. While trastuzumab seems to be effective only against breast cancer with amplified Her2 gene copy numbers and/or high Her2 surface expression, T-cells activated by tumor vaccines could potentially recognize tumors with intermediate or low levels of this molecule. Moreover, there is evidence that trastuzumab may synergize with specific T-cells [12], making a combinatorial approach with vaccination and trastuzumab an attractive clinical treatment modality.

pDNA immunization has several advantages as compared to other vaccination strategies; while immunization with proteins primarily induces antibody responses, pDNA vaccination efficiently promotes generation of antigen specific T-cells as well as antibody production [13]. Similarly, whereas peptide injections only activate the limited number of T-cells expressing corresponding T-cell receptors, pDNA immunization may activate immune responses to a broad repertoire of epitopes. Also, while peptide immunization could induce T-cell tolerance and thus enhanced tumor growth if not given with an efficient adjuvant, pDNA immunization ensures antigen-presentation by potent antigen presenting cells (APCs) [14]. Notably, the nucleotide sequences of pDNAs can themselves act as adjuvants [15], but the drawback of competing vector specific immunity associated with viral vaccines is circumvented [16]. Moreover, Her2-pDNA vaccination has been applied extensively in experimental models, where it induced protective immunity against transplantable tumors as well as against spontaneous tumor development in Her2-transgenic mice [11,17].

Since immunization of dogs with a human tyrosinase DNA vaccine produced clinically significant and durable responses [18,19], a conditional license has been issued for canine melanoma therapy by USDA - the regulatory agency of animal vaccines - as the first anti-cancer DNA vaccine strategy approved in any species in the USA [20]. Nevertheless, pDNA vaccination is often considered an ineffective approach for immunization in humans. Notably, vaccine efficacy in animal models has been improved by including cytokines or plasmids coding for these as adjuvants [21-24].

Here we present a pilot clinical trial to evaluate the safety and tolerability of a pDNA coding for a full-length Her2 molecule administered together with low-doses of the cytokines granulocyte macrophage colony stimulating factor (GM-CSF) and interleukin (IL)-2 in eight patients with metastatic breast carcinoma overexpressing Her2. All but one patient received concomitant trastuzumab treatment during the study period. This is the first report on administration of a Her2-pDNA vaccine in humans. We demonstrate that injection of the pDNA vaccine and cytokines during concurrent trastuzumab treatment was safe, well tolerated and induced specific endogenous antibody responses as well as late-onset CD4^+ ^T-cell responses in patients with advanced breast cancer.

## Patients, Materials and Methods

### Patient characteristics

The study was performed at the Oncology clinic, Radiumhemmet, Karolinska University Hospital, Stockholm, and was approved by the local ethics committees in Uppsala and Stockholm and the Swedish Medical Product Agency. Eight patients with histologically verified breast cancer with advanced/metastatic disease were included in the study, but only six completed three full vaccination cycles (see table 1 for summary of patient information). All patients received verbal and written information and were included after informed consent in accordance with the Declaration of Helsiniki. Eligibility criteria included a Zubrod/ECOG performance status of three or less and an expected survival of more than three months. Patients were receiving or had been offered standard-of-care therapy for Her2-overexpressing, locally advanced or metastatic breast carcinoma at the time of accrual. Her2 status was routinely determined by immunohistochemistry using the antibodies CB11 (Ventana, and from 2007 Novocastra Leica, Wetzal, Germany), A485 (DAKO, Glostrup, Denmark) and AB17 (Neomarkers, LabVision Freemont, CA, USA) between the year 2000 and March 2005, and only CB11 and A485 thereafter. The internal control constituted of four breast cancer cell lines exhibiting different Her2 positivity: BT474 (3+), MDA453 (2+), RT4 (1+), and 5637 (0). Moderate to strong Her2 stainings were verified by fluorescence *in situ *hybridization (FISH) to exclude false-positives, and gene amplification was demonstrated by inclusion of a centromere probe, according to the standard routines at Karolinska University Hospital.

**Table 1:** Patient characteristics

Patient #	Age [years]^ǂ^	Disease status	Site of metastasis	Previous treatments (abbreviations explained below)	ER/PR^▫^	Vaccine cycles	Side effects	Trastuzumab and pDNA vaccine concurrence	Survival [month] from diagnosis*,^+^	Survival [month] after first vaccine	Alive/dead at last follow up*
**1**	60	PD^Δ^	bone	Surgery, FEC, DO/T, T, RT, PA/T, VI/T, RT	-/-	3	-	No	46	9	Dead
**2**	44	PD	skin	FEC, surgery, FEC, RT, PA/T, DO/T, VI/T, CP/T, surgery, T	-/-	1	-	Yes	90	42	Dead
**3**	53	PD	Bone, LN°	surgery, T, CA/T, T	-/-	3	-	Yes	80	58.5	Alive
**4**	64	PD	LN	(surgery, 5-FU, OP, RT, FEC, DO, Platinol/CA, VI/T, PA/T, surgery	?	3	-	Yes	96	55	Alive
**5**	61	PD	Bone, lung, liver	Surgery, FEC, RT, TA, CA/T, PA/T, RT, T	+/+	1	-	Yes	62	14	Dead
**6**	64	PD	LN, liver	FEC, DO, surgery, RT, surgery, VI/T, CA/T	-/- +/+^∞^	3	-	Yes	72	21.5	Dead
**7**	47	PD	Liver, lung	Surgery, FEC, RT, TA/GHRH analog, DO/T, CA/T, IX, VI/T, T	-/+	3	-	Yes	58	6.5	Dead
**8**	67	PD	LN	Surgery, FEC, RT, TA, PA, DO, CA, CM/T/MT, CM/BV, VI/T	-/+	3	-	Yes	92.5	28	Dead

BV -- Bevacizumab, CA -- Capecitabine, CM -- Cyclophosphamide, CP -- Carboplatin, DO -- Docetaxel, 5-FU -- 5-Fluorouracil, FEC -- epirubicine, cyclosphamide, 5-FU, IX -- Ixabepilon, MT -- Methotrexat, OP -- Oxaliplatin, PA -- Paclitaxel, RT -- radiotherapy, T -- Trastuzumab, TA -- Tamoxifen, VI -- Vinorelbine

^ǂ ^at enrolment, ^Δ^PD -- progressive disease, ° LN -- lymph node, ^▫^ER/PR - estrogen/progesteron receptor, ^∞^expression site dependent,

*latest follow up July 2009, 87 month after study initiation, ^+^median survival 76 month

Of the six patients that completed the study, five were treated with trastuzumab throughout all three vaccination cycles and the remaining patient (patient #1) received trastuzumab prior to and again four months following vaccination. This variation in treatment was due to the fact that concomitant trastuzumab administration was allowed, but not an integrated part of the experimental treatment. Exclusion criteria included a significant history or evidence of cardiac disease including congestive heart failure, coronary artery disease, uncontrolled hypertension, serious arrhythmia or evidence of prior myocardial infarction on ECG, absence of measurable disease or evidence of current serious medical or psychiatric conditions, which would hinder informed consent or treatment.

### Design, construction and production of Her2-pDNA vaccine

To minimize the risk of malignant transformation of cells at the site of injection a kinase deficient Her2 DNA sequence (E2A) containing a mutation in codon 753 to convert a lysine (AAA) to an alanine (GCA) residue in the ATP binding site [25,26] was used. From the pCMV-E2A vector the E2A insert was subcloned into pVax1 (Invitrogen, Leek, The Netherlands) to generate pVaxE2A (Her2-pDNA) for clinical use. The correct sequence of pVaxE2A was verified by DNA sequencing.

The pVax1 vector complies with the Food and Drug Administration, Center for Biologics Evaluation and Research (FDA CBER) regulations for vectors to be used in human DNA vaccination protocols. The vaccine was produced by the "Gene Therapy Center" at Karolinska University Hospital Huddinge, Stockholm, under Good Manufacturing Practice (GMP) conditions with endotoxin content less than or equal to 10 EU/mg, >85% supercoiled plasmid DNA, protein content <10 μg/mL of plasmid and chromosomal DNA content <30 μg/mL of plasmid. The vaccine was aliquoted in saline solution, stored at -80°C and thawed immediately prior to administration. All handling of the DNA vaccine was performed according to national institute of health guidelines for research involving recombinant DNA molecules. Her2 protein expression was verified by flow cytometry after transfection of Cos7 cells with Her2-pDNA and by immunohistochemistry after intramuscular (i.m.) injection in mice (data not shown).

### Administration of the Her2-pDNA vaccine

The immunization protocol using the cytokines GM-CSF and IL-2 as adjuvants, was selected based on encouraging immunological responses in our previous pDNA trial using a similar administration schedule to target the prostate cancer antigen PSA in patients with hormone-refractory prostate cancer [27]. Also, we have shown that the same pVaxE2A Her2-pDNA construct as used in the vaccine can induce protective immunity in mice when co-injected with a GM-CSF encoding plasmid [28].

**Figure 1:**
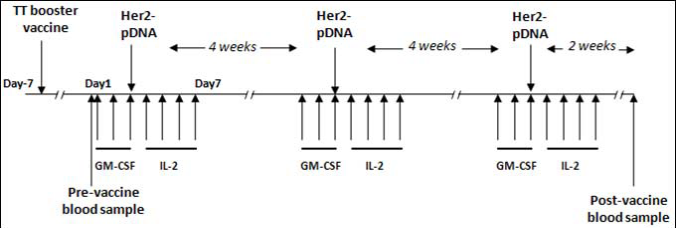
**Schematic overview of the Her2-pDNA vaccination schedule**.

The clinical protocol comprised three pDNA vaccination cycles per patient. In each cycle, Her2 plasmid was administered both i.m. (270 μg) and intra cutaneously (i.c.) (30 μg). Patients also received 3 daily i.c. injections of GM-CSF (40 μg Leukomax, Novartis, Basel, Switzerland) at the same location as the i.c. vaccine injection, starting two days prior to Her2-pDNA vaccine administration. Injections of low-dose IL-2 (1 μg/kg Proleukin, Prometheus Laboratories, San Diego, CA, USA) were given subcutaneously (s.c.) in the abdominal region for four consecutive days, starting 24 hours after the pDNA vaccination. A tetanus toxoid (TT) vaccination prior to Her2-pDNA vaccination was used as a control for immunomonitoring. Figure 1 provides an overview of the treatment schedule.

### Collection of blood samples and isolation of peripheral blood mononuclear cells (PBMC)

Blood and serum samples were collected by venipuncture from the patients immediately before the first and approximately two weeks after the last vaccine cycle. Three patients (patient #3, 4 and 8) were long term survivors and were followed up at a later time point (22, 38.5 and 41 months after last vaccination, respectively). PBMC were isolated by Ficoll-Hypaque (Amersham Biosciences, Uppsala, Sweden) density gradient centrifugation.

### Proliferation assay

T-cell proliferation was assessed using a modified limiting dilution assay shown to be useful for evaluation of low frequency T-cell responses [29]. Freshly isolated PBMC from patients at every time point and thawed PBMC from a healthy donor known to be reactive to TT and phytohemagglutinin (PHA) stimulation as an inter-experimental control were plated in 12-24 identical wells per stimuli in medium alone, or with 1 μg/mL recombinant human Her2 protein (a kind gift from Dr. Catherine Gerard, GlaxoSmithKline Biologicals, Belgium), 5 μg/mL TT (Tetravac, Sanofi Pasteur MSD, Brussels, Belgium) or 5 μg/mL PHA (Sigma-Aldrich, Irvine, UK). On day four 1 μCi [methyl-3H]-Thymidine (Amersham Biosciences, Freiburg, Germany) per well was added. 24 h later plates were harvested and measured using a scintillation counter (1450 MicroBeta, Trilux, Wallac, Turku, Finland).

A standard stimulation index (SSI) ≥ 2, defined as at least twice the mean cpm in stimulated wells compared to the mean cpm of control wells, was considered as antigen specific proliferation. The percentage of wells exhibiting [methyl-^3^H]-Thymidine uptake greater than the mean plus three standard deviations of the corresponding wells cultured with media alone served as an additional semi-quantitative measure of responding T-cells [30].

### Her2-specific interferon (IFN)-γ ELISpot

Four Her2-derived peptides were used to detect CD4^+ ^T-cell responses in enzyme-linked immunospot (ELISpot) assays (Mabtech, Nacka Strand, Sweden) as previously described [31,32]. Each of these recently identified 15-mer peptides p59, p88, p422 and p885 [33] (designated by the position of the first amino acid in the Her2 protein) were in computer modelling predicted to bind multiple human leukocyte antigen (HLA)-DR molecules and indeed found to exhibit high-affinity binding to a variety of major histocompatibility complex (MHC) class II [33,34]. Pooled cytomegalovirus, Epstein-Barr virus, and Influenza viral peptide epitopes (CEF, Mabtech, Nacka Strand, Sweden) were used as positive control.

A positive response was defined as the peptide-specific spot number that was significantly higher (triplicates) than control wells using a two-tailed *t *test (*P *< 0.05). Counts for each peptide were tallied and reported as the total number of Her2-specific T-cells assessed at each time point. It may be possible that while the peptides bound multiple HLA-DR alleles, some of them could additionally contain embedded motifs that could stimulate CD8^+ ^T-cells. However, Her2-specific CD8^+ ^T-cell responses are typically lower by at least one order of magnitude even in vaccinated patients [31]. Putative CD8^+ ^responses against p369, p435 and p689 9-mer peptides known to bind to HLA-A2 were tested (data not shown), but are of limited value since patients were not HLA-typed. Changes between pre- and post-immunization responses were considered significant if there was at least a two-fold increase or a 50% decrease.

### Enzyme-linked immunosorbent assay (ELISA)

ELISAs measuring amounts of Her2-specific Ig λ antibodies have been previously described [35]. TT ELISAs served as internal controls.

All serologic assays were repeated at least twice for each individual patient. A humoral response was considered positive by a relative A450 index of >2 or a titer <1/100.

### Statistical analysis

Statistical analyses were performed using Excel, GraphPad, InStat or Prism Software (GraphPad Software, Inc, La Jolla, CA USA). Data were analyzed using two-tailed Mann-Whitney (nonparametric data) or Student's *t *tests unless otherwise stated, and the results were considered statistically significant if p < 0.05.

## Results

### Patient characteristics and clinical observations

Eight women with a mean age of 57.5 years were accrued in this study. Patient characteristics are summarized in Table 1. All patients had advanced breast cancer treated with extensive prior therapy, including trastuzumab. All patients except one (patient #1) were on trastuzumab treatment during the study period.

Of the eight patients entering the trial, six completed all three vaccination cycles. Patient #2 was withdrawn after one cycle due to severe erysipelas at the location of a skin metastasis and patient #5 due to disease progression. No significant side effects associated with the vaccination or cytokine administration were observed in any patient. There were no manifestations of autoimmunity or cardiotoxicity, nor was any acute toxicity observed.

Of the six patients that completed all three cycles of vaccination, two were long term survivors, still alive more than 4 years after the last vaccination (in July 2009 >56 months for patient #3 and >53 months for patient #4). Patient #8 lived until 25 month post vaccination. The median survival time from diagnosis to latest follow up for all 8 enrolled patients was 76 months with a range of 46-96 months.

### Evaluation of Her2-specific T-cell responses

Lymphocyte proliferation assays were performed with freshly isolated PBMC from pre- and post-vaccination blood samples of all patients. As expected, PHA induced significant proliferation in all tested wells with an average SSI of 69.6 across pre- and post-Her2 vaccination assays. Importantly, vaccine-induced TT-specific T-cells proliferated upon stimulation with cognate antigen in 100% of the wells. The average SSI was similar in pre-Her2-vaccination (15.8) and post-Her2-vaccination (13.9) samples, indicating that the TT booster vaccination resulted in stable cellular immunity to TT over the treatment period. In contrast, the overall proliferative responses to Her2 protein were minimal as average SSIs were negative and the percentage of wells with positive proliferative responses were very low in both pre- (SSI 1.0, range 0.8-1.3; 1.8% of wells exhibiting Her2-specific proliferation) and post- (SSI 1.0, range 0.9-1.1; 3.3% of wells exhibiting Her2-specific proliferation) Her2 vaccination samples. The cut offs in mean stimulation index (SI) for scoring individual experimental wells as responding or non-responding was on average 1.91 (range 1.4 - 2.4), which is in line with previous reports [29,36]. Although the frequency of wells exhibiting significant proliferation to Her2 protein was almost twice as high after the treatment regimen, the average SIs of the positive wells in the post-vaccination samples (2.1) was only about half that of the pre-vaccine samples (4.0). Thus, weak and rare pre-existing Her2 protein specific proliferative responses were observed in fresh PBMC, but these responses were not significantly enhanced after the Her2-vaccination regimen.

**Figure 2:**
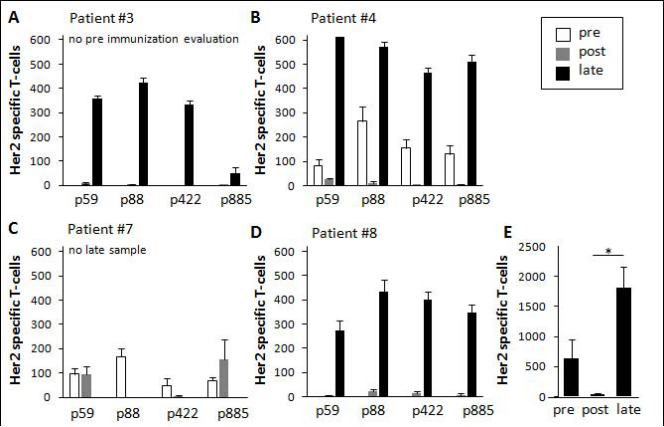
**MHC class II restricted T-cell responses to Her2 before and after Her2-pDNA vaccination**. **A-D**. Her2-specific IFN-γ production by T-cells from patients #3, 4, 7 and 8, before (3 days pre-) and after (10 days post-) Her2-pDNA vaccination and at long term follow-up (41, 38.5 and 22 month after last vaccination for patients #3, 4, 8, respectively). Bars show mean (± s.e.m.) frequency of IFN-γ producing T-cells (spot forming units) per 2.5 × 10^5 ^PBMC responding to a panel of 4 degenerate Her2-derived HLA-DR epitopes (p59, p88, p422 and p885). No pre-bleeding ELISpot was performed for patient 3 due to insufficient numbers of available PBMC. **E**. Mean ± s.e.m. Her2-specific T-cell frequency per 2.5 × 10^5 ^PBMC in patients evaluable at all time points. Bars show pooled responses of patients #4 and 8 pre, post and late. *: p ≤ 0.05.

For four of the patients that completed all three vaccine cycles, sufficient amounts of PBMC were available to evaluate Her2-specific cellular immunity towards a panel of HLA-DR restricted peptides by ELISpot. Two of three evaluable patients (patients #4 and 7) demonstrated pre-vaccination CD4^+ ^T-cell mediated immunity to all four Her2-derived peptides, while no pre-vaccine immunity to these epitopes could be detected in patient #8 (Figure 2). No pre-vaccine ELISpot could be performed for patient #3 due to paucity of PBMC.

For the patients who had samples permitting pre- versus post-vaccination comparison (patients #4, 7 and 8), there was no consistent change in peptide specific responses resulting from immunization when tested 10 days after the last vaccination. Intra-patient comparison of pre- and post-vaccination responses to individual peptides showed that both boosting and reduction of pre-existing responses occurred and also that new T-cell specificities could be induced by the treatment (Figure 2A-D). Interestingly, increased, decreased and unchanged responses to individual peptides could be observed in the same patient, e.g. patient #7, after three cycles of Her2-pDNA vaccination (Figure 2C).

Three of the four patients (patients # 3, 4 and 8) survived more than two years after the last vaccination and an additional blood sample was collected from each of these subjects at a later time point. Strikingly, PBMC from all three patients exhibited strong Her2-specific immune response against all tested peptides at this late follow up. The frequency of Her2-specific T-cells was significantly increased compared to both pre- and post-vaccination samples in all patients and newly induced responses as well as recovery of responses lost at post-vaccination evaluation were observed. Individual results and pooled responses from patients evaluable at all time points (Figure 2E) show a significant increase of MHC class II restricted T-cell responses to Her2-derived epitopes at long term follow-up, while there was a transient decrease in Her2-specific immunity immediately after three cycles of Her2-pDNA plus GM-CSF and IL-2.

### Evaluation of Her2-specific antibody responses

**Figure 3:**
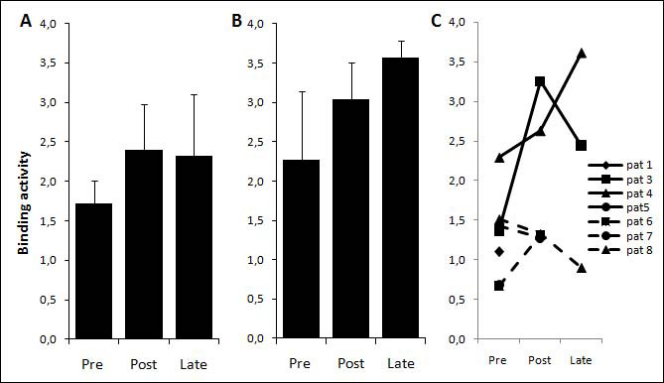
**Her2-pDNA vaccination generates Her2-specific humoral immunity**. **A-B**. Mean binding activity derived from **A**. Her2-specific or **B**. tetanus toxoid Ig λ-subclass specific ELISAs. Bars show the mean (± s.e.m.) binding activity of patients evaluable at all time points (patient #3, 4, 8, pre, post and late). **C**. Binding activity in the serum of all patients at all available individual time points (pre- and post-immunization as well as long-term follow up at 22-41 months following the last immunization).

Pre- and post-vaccination sera from all patients were analyzed for the presence of anti-Her2 antibodies. Since most patients received concurrent trastuzumab treatment during the Her2-pDNA vaccinations, λ-subclass specific ELISAs were performed. The specific detection of λ-subclass anti-Her2 antibodies allowed measurement of endogenous antibody production without detection of trastuzumab, an IgG1κ antibody present at high serum concentrations during therapeutic administration [37]. Comparison of pre- and post-Her2-pDNA vaccine responses in patients evaluable at all time points showed a trend towards higher mean binding activity of post- versus pre-vaccination sera against Her2 (Figure 3A). Notably, the Her2-specific binding activity in the responding patients reached levels comparable to those of the TT-specific antibodies following TT vaccine administered as a control before the Her2-pDNA vaccination schedule (Figure 3B, C). One of eight (12.5%) patients enrolled in the study had a pre-existing antibody response against Her2, as defined by a binding activity >2. The majority of evaluable patients (3/5) showed an increased Her2-specific binding activity after completion of three vaccination cycles (Figure 3C).

Two out of three patients that were available for long term monitoring could sustain their positive post-vaccination antibody levels for several years after the last vaccination. These two patients were also the ones that reached positive anti-Her2 binding activity that could be measured after 3 cycles of vaccination. The third long term surviving patient (patient #8) never exhibited any Her2-specific humoral immunity (Figure 3C).

## Discussion

From this small pilot study we can conclude that our full length Her2-pDNA, administered together with GM-CSF and IL-2, is safe, well tolerated and can induce both antibody and T-cell responses in advanced stage cancer patients. Since our group and others have shown that Her2 can down-modulate MHC class I expression [38-40], tumor vaccine strategies such as pDNA administration that are not solely dependent on CTLs but induce an integrated immune response involving also antibodies and CD4^+ ^T-cells should be advantageous. Bolstering this hypothesis is the observation that the same pDNA vaccine as used in the present trial can efficiently induce Her2-specific antibodies as well as a CD8^+ ^T-cell response and protection from tumor challenge in conventional and human Her2-transgenic BALB/c and HLA-A2 transgenic B6 mice [25,38].

The present clinical trial is the first to combine a Her2-pDNA vaccine with trastuzumab treatment. In light of preclinical studies demonstrating that tumor cells binding trastuzumab were more efficiently recognized by Her2 reactive T-cells [12], concomitant administration of trastuzumab and Her2 vaccines may cause substantial synergies and represents a promising treatment strategy. Combination therapy with trastuzumab and a peptide (E75) vaccine was recently applied in a subset of seven strongly Her2-positive cancers where this combination proved to be safe and immunologically beneficial [41]. A similar conclusion was reached for a Her2 T-helper peptide-based vaccine in combination with trastuzumab [42].

The combinatorial treatment complicated our attempts to detect vaccine-induced Her2-specific antibodies in the vaccinated patients. However, a recently established λ-subclass specific ELISA allowed evaluation of endogenous Her2-specific antibody responses without detection of or interference by the IgG1κ antibody trastuzumab [35]. Notably, the majority of evaluable patients demonstrated increased antibody binding activity after completion of the vaccine trial and in most of the long term survivors these endogenous Her2-specific antibodies persisted or were increased in samples obtained several years after the last vaccine administration. Due to co-administration of trastuzumab we were not able to evaluate the contribution of endogenous Her2-specific antibodies of the κ-subclass to the overall humoral immune response. Considering previous vaccine trials [43] it is likely that IgG κ antibodies were also induced.

The ability of our vaccine to trigger Her2-specific antibody responses has significant therapeutic implications, as a broader repertoire of Her2-reactivities and antibody isotypes may lead to enhanced tumor specific antibody dependent cellular cytotoxicity or enhanced antibody-induced perturbation of Her2 signaling. Similarly, it is possible that the endogenously induced antibodies synergize with trastuzumab or are more efficient in opsonizing Her2 expressing tumor cells or fragments of these, leading to better uptake by APCs and thus improved activation of endogenous T-cells. Also, numerous mouse models have implicated vaccine induced antibodies as a major factor in conferring protection against transplantable and spontaneous Her2 expressing tumors [44-46].

Mainly non-professional APC in PBMC were available to process and present epitopes to T-cells in our proliferation assays. This may have prevented detection of rare and/or weak autologous T-cell responses to the recombinant Her2 protein, while allowing strong TT-peptide specific T-cell responses to be readily detected. Indeed, most evaluated patients showed pre-vaccination CD4^+ ^T-cell reactivity to all tested peptides in IFN-γ ELISpot assays against Her2-derived 15-mer peptides known to bind several different HLA-DR allotypes [34]. However, for the three patients who had samples that allowed a pre- versus post-vaccination comparison, we failed to observe a consistent increase in peptide specific CD4^+ ^T-cell responses. In the event that Her2-specific immune responses were induced or boosted, activated T-cells may have homed to the site of the tumor, hampering their detection in peripheral blood. Alternatively, one may speculate whether the induction of regulatory T-cells by the IL-2 [47], and/or induction of myeloid derived suppressor cells by the GM-CSF [48] in our treatment regimen may account for the lack or decrease in immune responsiveness and the almost complete disappearance of pre-vaccination immunity against all four tested epitopes in one patient. Regrettably, the reason could not be experimentally established due to paucity of patient PBMC.

In contrast to the absence of CD4^+ ^T-cell responses early after vaccination, the three patients who survived more than two years after the last vaccination all exhibited strong immunity to all of the tested Her2-derived peptides when re-evaluated at a late time point. This late immune response to Her2 following vaccination is not without precedence. Morse *et al. *[49] provided evidence that the peak response to a DC vaccine loaded with Her2 intracellular domain could occur more than 5 years after concluding vaccine therapy, and Disis and colleagues [43] showed that anti-Her2 T-cell responses could persist for at least 1 year after vaccination with T-helper epitope derived peptides mixed with GM-CSF had ended. Since Her2-specific antibody and T-cell responses have also been detected in non-vaccinated patients [35,50], and were further confirmed in the pre-treatment samples in the present study, we cannot exclude that this late response is unrelated to the vaccine administration and instead induced by the trastuzumab therapy [35] or by patients' Her2 expressing tumors.

Although Her2 is overexpressed in a broad range of carcinomas, low levels are also present in normal epithelial surfaces [51]. The concern is therefore that induction of an immune response to this "self-antigen" should lead to autoimmune manifestations. Alternatively, since trastuzumab can induce cardiac toxicity in a small but significant proportion of treated patients [52], one may consider whether the endogenously-induced Her2-specific antibodies reported in this study and by others may contribute to or worsen this side effect. It is therefore important to note that none of the eight patients who received the Her2 vaccine had any manifestations of autoimmunity or cardiac toxicity. This is in concordance with observations in other Her2 vaccine trials in which no adverse effects have been reported [43,49]. This includes a trial based on the E75 peptide derived from the extracellular domain of Her2 and GM-CSF, which resulted in a decreased disease recurrence rate [53].

Since this trial was a small phase I clinical study with only six patients completing all three cycles of vaccine and cytokine administration, this precludes any conclusion regarding the clinical efficacy. Further complicating interpretations of clinical efficacy, all patients suffered from advanced disease and had undergone prior chemotherapy and most were on concomitant trastuzumab treatment. Nevertheless, it is noteworthy that three of the six patients who received all three cycles of vaccine treatment were long-term survivors. The median overall survival from start of vaccination was 24.8 months, with a range of 6.5 to 58.5 months, but as mentioned the significance of these data must be interpreted with caution because of the small patient number.

The median survival for patients in a randomized study failing first line trastuzumab therapy was 25.5 months for patients receiving continuous trastuzumab combined with capecitabine [54]. In another randomized study patients who failed conventional chemotherapy-trastuzumab combinations had an estimated median survival of about 58 weeks on the combination of lapatanib and capacitabine [55].

The relatively long survival from the start of vaccination for patients #3 and #4, 58.5 and 55 months, respectively, is obviously an interesting observation, especially as broad Her2-specific immunity was detected in these patients. However, these two patients had disease burden limited to lymph nodes and skeleton when entering the study and long term survival in this category of patients is not unusual. Patient #4 nevertheless had failed several lines of therapy before inclusion, indicative of treatment-refractory disease, but continued to be treated with trastuzumab as single agent after the end of vaccination.

## Conclusion

Our pilot study demonstrates the feasibility, safety and tolerability of Her2-pDNA vaccination in combination with GM-CSF and IL-2 in a small number of advanced breast cancer patients who are on concurrent trastuzumab treatment with findings warranting further exploration of this concept. The induction of long-lasting cellular and humoral immune responses against Her2 are encouraging and occasional patients appear to draw clinical benefit from this treatment, although this must be confirmed in further studies, at best with a randomized design. Her2-pDNA vaccines already provide a promising strategy by broadening or potentiating the response to trastuzumab administration, which is now a standard adjuvant therapy for women with Her2 overexpressing breast cancer. If our and similar vaccine strategies efficiently generate humoral Her2-specific responses, trastuzumab may later become obsolete and vaccines alone successful against early and metastatic breast cancer. This would facilitate the practical management of Her2 positive carcinomas, since trastuzumab based strategies are expensive and require time-consuming three-weekly intravenous administrations. If demonstrated to have a favorable benefit-risk ratio the vaccination approach should also be studied as a preventive strategy in high risk individuals.

## Competing interests

The authors declare that they have no competing interests.

## Authors' contributions

HN designed and performed research, analyzed data and performed statistical analysis. He was together with IP responsible for collecting and handling patient samples and for performing the T-cell proliferation assays and early attempts to measure specific antibody responses. IP performed research, analyzed data and drafted the manuscript. She was responsible for collecting and handling the patient samples after HN had departed from CCK and co-ordinating the collaboration with KLK's laboratory. Together with EL she also summarized and processed the patient data and together with RK she wrote the manuscript. JC designed research. He was involved in writing the clinical protocol and the early phases of the study. WZW contributed new reagents/analytic tools. She provided the vaccine construct and was responsible for the pre-clinical testing of this vaccine in mouse models. CE performed research by being responsible for performing the ELISA and ELISPOT assays. MPP contributed new reagents/analytic tools by collaborating with WZW in the pre-clinical testing of the vaccine in mouse models. KLK designed research, analyzed data and performed statistical analysis. He was responsible for the design of the ELISA and ELISPOT assays and the testing of patient samples in these assays was carried out and interpreted in his laboratory with the assistance of CE. JB designed research and provided expert opinion for the study. He was the principal investigator of the clinical study and responsible for the contact with the regulatory agents. EL performed clinical research. She was the physician who had all patient contact and thus carried out all vaccination procedures and also summarized the patient information for the manuscript. RK designed research, analyzed data and wrote the paper. He initiated and designed the study and wrote the clinical protocol. He funded all costs involved and was responsible for the immune monitoring, with input also from the lab of KLK. All authors read and approved the final manuscript.
